# Magnesium supplements, sleep quality, and nocturnal leg cramps: a combination of cross-sectional and prospective studies

**DOI:** 10.1007/s00394-026-03910-2

**Published:** 2026-02-16

**Authors:** Noelle Stadie, Raphaël Heinzer, Pedro Marques-Vidal

**Affiliations:** 1 University of Lausanne, Lausanne, Switzerland; 2 Centre of Investigation and Research on Sleep (CIRS), Lausanne University Hospital (CHUV) and University of Lausanne (UNIL), Lausanne, Switzerland; 3 Department of Medicine, Internal Medicine, Lausanne University Hospital (CHUV) and University of Lausanne (UNIL), Office BH10-642, Rue du Bugnon 46, 1011 Lausanne, Switzerland

**Keywords:** Sleep, Night cramps, Magnesium, Cross-sectional study, Prospective study

## Abstract

**Purpose:**

Many individuals turn to supplements like magnesium, believing them to offer a natural way to improve sleep quality. Yet, research on magnesium’s effects has produced inconsistent results. We assessed the relationships between magnesium supplementation and subjective/ or objective sleep parameters, including night cramps.

**Methods:**

Data from three follow-ups of the population based CoLaus|PsyColaus cohort: 2009–12 (first), 2014–17 (second), and 2018–21 (third). Magnesium supplement use was self-reported, and participants were categorized either as users or non-users. Subjective sleep parameters were measured through questionnaires, and objective sleep parameters with polysomnography. The researchers computed a polygenic risk score (PRS) including five single nucleotide polymorphisms related to magnesium levels.

**Results:**

The number and characteristics of the participants in the first, second, and third follow-ups were 3887 (52.6% women, 57.2 ± 10.4 years, 4.5% magnesium users), 1916 (49.7% women, 60.8 ± 9.4 years, 7.9% users), and 1561 (51.2% women, 63.1 ± 8.9 years, 7.5% users), respectively. The multivariable-adjusted analyses indicated that magnesium users had a higher likelihood of experiencing night cramps or restless leg syndrome, with a trend towards poorer sleep quality. Multivariable-adjusted analyses revealed no significant differences between participants with and without cramps regarding polysomnography results or magnesium PRS. The prospective analyses demonstrated that participants taking magnesium supplements at baseline had a higher incidence of night cramps. The incidence of restless leg syndrome did not differ between users and non-users.

**Conclusion:**

We found no consistent association between magnesium supplementation and subjective or objective sleep parameters or restless leg syndrome. Magnesium supplementation did not prevent night cramps.

**Graphical abstract:**

**Figure:**
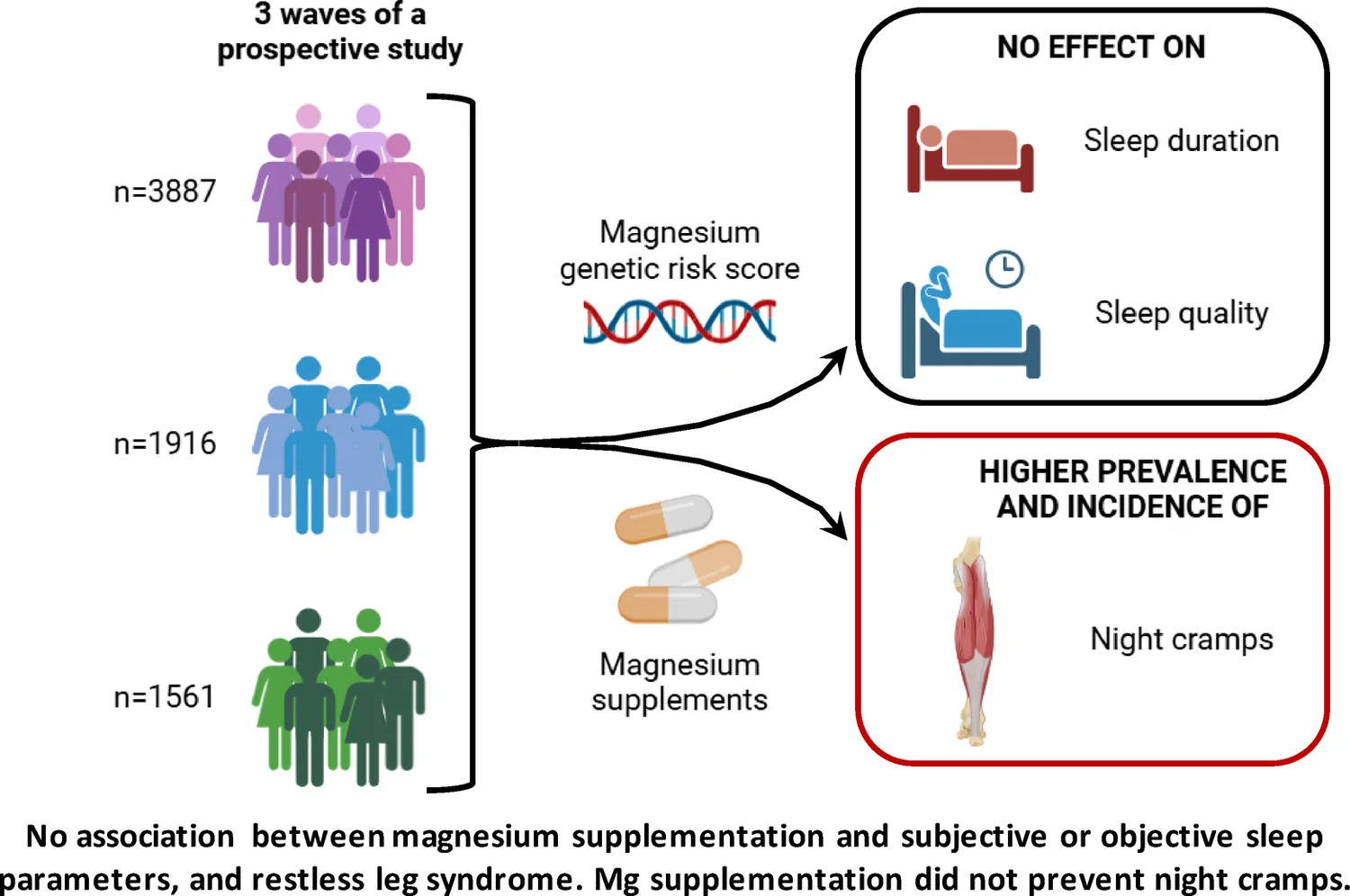

**Supplementary Information:**

The online version contains supplementary material available at https://doi.org/10.1007/s00394-026-03910-2.

## Introduction

Sleep is an essential component of overall health and wellbeing, deeply intertwined with various physiological and cognitive functions. As sleep disorders and poor sleep quality become more prevalent in modern societies [1–3], their impact on both physical and mental health is increasingly evident. In response, many individuals have turned to vitamin supplements, believing them to be a natural means of enhancing sleep quality and achieving more restorative rest. In fact, a significant portion of the Swiss population consumes vitamin/mineral supplements [4], largely due to the belief that they provide various health benefits, although the scientific evidence supporting these claims remains limited.

Magnesium is one mineral supplement that is believed to be beneficial regarding sleep. Research about the association between sleep parameters and magnesium has shown mixed results. While an interventional study reported improvements in sleep quality [5], findings from randomized controlled trials (RCTs) were inconsistent [6], with a majority showing no significant effects. This lack of notable impact was echoed in a systematic review with meta-analysis [7]. Another study suggested that low blood levels of magnesium and calcium, but not vitamin D, might be linked to poor sleep quality in airline pilots, a population that often experiences sleep disturbances due to irregular schedules [8]. The results of this study demonstrated contradictory findings about whether magnesium could help in treating nocturnal muscle cramps or not. Many reviews showed no significant effect [9–11], while also claiming that they could have been underpowered [12] or that the studies lacked robustness [13]. Notably, most studies about magnesium either had a small sample size or only investigated a specific population (ex: air pilots [8], elderly people [7], or people needing haemodialysis [13]), so it could be interesting to see whether the results also apply to the general population.

Additionally, it is known that several genetic variants influence blood magnesium levels. Therefore, it could be interesting to investigate whether people who have single nucleotide polymorphisms (SNP’s) linked to lower magnesium levels have more cramps than people having SNP’s coding for higher magnesium levels. Still, to our knowledge, no study has ever assessed the association between genetic markers of magnesium levels with cramps/restless leg syndrome or other sleep characteristics.

Hence, the objective of this study was to assess the potential associations between magnesium supplement use and both subjective and objective sleep parameters, namely night cramps and restless legs syndrome, using data from a population-based study conducted in Lausanne, Switzerland. Our hypothesis was that magnesium supplements are not associated with sleep parameters, night cramps or restless legs syndrome.

## Material and methods

### Study setting

The CoLaus|PsyCoLaus study is a population-based study investigating the epidemiology and genetic determinants of psychiatric and cardiovascular disease in Lausanne, Switzerland [14]. Briefly, a representative sample was collected through a simple, non-stratified random sampling among Lausanne inhabitants aged between 35 and 75. The baseline study was conducted between June 2003 and May 2006; the first follow-up was performed between April 2009 and September 2012; the second follow-up was performed between May 2014 and April 2017 and the third follow-up was performed between April 2018 and May 2021. As information on sleep was only collected during the follow-ups, only data from those periods was used.

### Main outcome: sleep questionnaires

Sleep quality was assessed with the Pittsburgh sleep quality index (PSQI). The PSQI is used to assess sleep patterns over the past month and can be completed by the rater alone or with a sleeping partner. The score varies between 0 and 21; indicative of overall sleep quality, as well as subscale values, including subjective sleep quality, sleep-onset latency, sleep duration, habitual sleep efficiency, sleep disturbances, use of sleep medication, and daytime dysfunction. A score > 5 indicates poor sleep quality [15].

Excessive daytime sleepiness (EDS) was assessed using the Epworth Sleepiness Scale (ESS). The score on this eight-item scale varies between 0 and 24; a score > 10 is commonly used to indicate EDS [16].

The probability of sleep disordered breathing (SDB) was assessed with the Berlin questionnaire (BQ) [17]. The BQ incorporates questions about snoring (category 1), daytime somnolence (category 2), and hypertension and body mass index (BMI) (category 3). Patients were scored as being at high-risk for SDB if they had a positive score on two or more categories [17]. We also used the NoSAS score [18] to assess risk of clinically significant sleep-disordered breathing. The NoSAS score ranges from 0 to 17, allocates 4 points for having a neck circumference > 40 cm, 3 points for having a BMI ≥ of 25 kg/m^2^ to < 30 kg/m^2^ or 5 points for having a BMI ≥ 30 kg/m^2^, 2 points for snoring, 4 points for being > 55 years of age, and 2 points for being male (Supplementary Table 1).

Obstructive sleep apnoea (OSA) severity was assessed using the STOP-BANG questionnaire [19]. It is composed of 8 questions requiring a yes/no response, and its value ranges from 0 to 8. The first component (STOP) includes questions on Snoring, Tired/sleepiness, Observed apnoeas, and blood Pressure. The second component (BANG) includes BMI, Age, Neck circumference, and Gender. A STOP score ≥ 3 indicates a high risk of OSA.

Restless legs syndrome (RLS) was assessed using the Restless Legs Syndrome Study Group criteria [20]. Presence of RLS was defined by positive responses to all four questions defined by the 1995 International RLS study group [20], see Supplementary Table 2.

Night cramps were extracted from item 7 of the Munich Parasomnia Screening (MUPS) [21] and categorized as present if the participants reported a frequency of at least one night with cramps per week.

Incidence of RLS and night cramps was assessed between the first and the second follow-ups among participants devoid of the condition at the first follow-up.

### Secondary outcome: polysomnography

The HypnoLaus sleep cohort study, [22] a nested-study of CoLaus|PsyCoLaus, was designed to assess the prevalence and correlates of sleep-disordered breathing in a general unselected middle- to-older-age population of Lausanne, Switzerland (September 2009 to June 2013). Details of sampling and procedure methodologies have been described elsewhere [22]. Briefly, 2,162 participants underwent a complete examination including demographic, clinical assessment, and an overnight unattended full polysomnography (PSG).

Participants were invited to attend the Centre for Investigation and Research in Sleep at the Lausanne University Hospital (CHUV, Lausanne, Switzerland) to be equipped with a polysomnographic recorder (Titanium, Embla Flaga, Reykjavik, Iceland) and undergo a full-night PSG at their home. PSG setup specifications followed the 2007 American Academy Sleep Medicine (AASM) recommendations. Physiological parameters evaluated during PSG recordings were electroencephalogram (central, occipital and frontal), electrooculogram (right and left eyes), surface electromyogram (chin and anterior tibialis muscle), electrocardiogram, airflow (nasal cannula), abdominal and thoracic respiratory efforts (respiratory inductance plethysmography), snoring and body position. In this study, the following parameters were considered: sleep onset (minutes), total sleep time (in minutes), sleep efficiency (%), number of awakenings, percentage of total sleep time spent in stage 1 sleep, stage 2 sleep, slow wave sleep and rapid eye movement (REM) sleep.

Periodic leg movements (PLMs) were scored according to the official World Association of Sleep Medicine standards [23] by the following criteria: (1) duration between 0.5 and 10 s, (2) minimum amplitude > 8 μV in electromyography (EMG) voltage above resting EMG, (3) end of the event when the EMG decreased to < 2 μV above the resting level and remained below that value for 0.5 s, (4) interval between 5 and 90 s between PLM onsets, (5) movements had to be part of a series of ≥ 4 consecutive movements meeting these criteria, and (6) PLMs on 2 different legs separated by < 5 s between movement onsets were counted as a single PLM. PLMs were not scored as PLMS if occurring at the end (± 0.5 s) of a respiratory event, defined as the beginning of the first breath that approximated the baseline breathing amplitude. The PLMS index (PLMSI) was calculated by dividing the total number of PLMS by total sleep time in hours.

### Exposure: magnesium supplement consumption

Participants reported all medicines and vitamin or dietary supplements prescribed or bought over the counter for the three follow-ups. All medicines and supplements were assessed and coded according to the Anatomic, Therapeutic and Chemical (ATC) classification of the World Health Organization (WHO). Magnesium supplements were considered if the ATC code started with A12CC (Magnesium, combinations with subtherapeutic amounts of vitamins). If the supplements were not listed in the Swiss compendium, further searches on the internet were conducted. Due to inaccurate reporting (i.e., reporting “Magnesium from the supermarket”), it was not possible to collect information regarding dose and duration of supplementation. Other vitamin (i.e., vitamin C alone or vitamin D + calcium) supplements devoid of Mg were also collected.

### Genotyping

Genome-wide genotyping was performed at baseline (2003–2006) using the Affymetrix 500 K SNP array. Nuclear DNA was extracted from the whole blood of participants considered of Caucasian origin, defined as having all four grandparents born in a selected number of countries. Genotypes were called using BRLMM [24]. Duplicate individuals, and first- and second-degree relatives, were identified and then removed by computing estimates pair-wise genomic kinship coefficients, using KING [25]. Subjects were excluded from the analysis in case of inconsistency between sex and genetic data, a genotype call rate < 90%, or inconsistencies of genotyping results in duplicate samples. Quality control for SNPs was performed using the following criteria: monomorphic (or with minor allele frequency < 1%), call rates < 90%, deviation from the Hardy–Weinberg equilibrium (p < 1 × 10^–6^). Phased haplotypes were generated using SHAPEIT2 [26, 27]. Imputation was performed using minimac3 [28] and the Haplotype Reference Consortium (HRC version r1.1) [29] hosted on the Michigan Imputation Server [28].

SNPs related to magnesium levels were selected from an online database (https://www.ebi.ac.uk/gwas/efotraits/EFO_0004845, assessed 17.10.2024). Five of the seven SNPs most significantly associated with magnesium measurements could be extracted from the genetic information available (Supplementary Table 3) and a polygenic risk score (PRS) was computed by adding the number of alleles associated with higher magnesium levels.

### Covariates

Smoking was self-reported and categorized as never, former (irrespective of the time since quitting smoking) and current. Education was categorized into high (university), middle (high school) and low (apprenticeship + mandatory). Marital status was defined as living alone (single, divorced, widowed) or living with a partner. Nationality was defined as Swiss or other. A standard drink was defined as a glass of wine, a bottle of beer, or a shot of spirits, approximating 10 to 12 g of ethanol [30]. Alcohol consumption was defined according to the number of drinks per week: 0, 1–13, 14–27, and ≥ 28 drinks/week.

Dietary intake was assessed using a self-administered, semi-quantitative food frequency questionnaire (FFQ) which also included portion size [31]. Briefly, this FFQ assesses the dietary intake of the previous 4 weeks and consists of 97 different food items. For each item, consumption frequencies ranging from “less than once during the last 4 weeks” to “2 or more times per day” were provided, and the participants also indicated the average serving size (smaller, equal or bigger) compared to a reference size. Each participant brought along her/his filled-in FFQ, which was checked for completion by trained interviewers the day of the visit. The alternative healthy eating index (AHEI) was adapted from McCullough et al. [32]. In our study, the amount of trans fat could not be assessed, and we considered all participants taking multivitamins as taking them for a duration ≥ 5 years. Thus, the modified AHEI score ranged between 2.5 and 77.5, instead of 2.5 and 87.5 for the original AHEI score [32]. It was not possible to extract Mg intake from the FFQ.

Body weight and height were measured with participants barefoot and in light indoor clothes. Body weight was measured in kilograms to the nearest 100 g using a Seca® scale (Hamburg, Germany). Height was measured to the nearest 5 mm using a Seca® (Hamburg, Germany) height gauge. Body mass index was calculated and categorized as normal (< 25 kg/m^2^), overweight (≥ 25 and < 30 kg/m^2^) and obese (≥ 30 kg/m^2^). As the number of participants with underweight (< 18.5 kg/m^2^) was small, namely among those taking Mg supplements: 2 in the first follow-up, 5 in the second, and 5 in the third, those participants were included in the normal weight category.

Blood pressure was measured using an Omron® HEM-907 automated oscillometric sphygmomanometer after at least a 10-min rest in a seated position, and the average of the last two measurements was used. Hypertension was defined by a systolic blood pressure (SBP) ≥ 140 mm Hg or a diastolic blood pressure (DBP) ≥ 90 mm Hg or presence of antihypertensive drug treatment.

Fasting plasma glucose was assessed by glucose dehydrogenase, with maximum inter and intra-batch CVs of 2.1% and 1.0%, respectively. Diabetes mellitus was defined as fasting plasma glucose ≥ 7.0 mmol/L and/or the presence of oral hypoglycaemic or insulin treatment. Sleep medications were considered for ATC codes beginning with N05C (hypnotics and sedatives) or N05BA (benzodiazepine derivatives). In the second and third follow-ups, blood magnesium levels were assessed after an overnight fast using xylidyle blue.

### Inclusion and exclusion criteria

For the cross-sectional analyses, participants were considered as eligible if they participated in one of the follow-ups. Participants were excluded if they had no data for (1) sleep; (2) any covariate. For the prospective analysis, participants were considered as eligible if they participated in the first follow-up. Participants were excluded if they had (1) previous RLS or night cramps; (2) no follow-up or no data at follow-up; (3) no data for any covariate.

### Ethical statement

The institutional Ethics Committee of the University of Lausanne, which afterwards became the Ethics Commission of Canton Vaud (www.cer-vd.ch) approved the CoLaus-PsyCoLaus study (project number PB_2018-00038, reference 239/09). All participants gave their signed informed consent before entering the study.

### Statistical analysis

Statistical analysis was conducted using Stata version 18.0 for Windows (Stata Corp, College Station, TX, USA). For bivariate analysis, results were expressed as number of participants (percentage) for categorical variables and as average ± standard deviation or median [interquartile range] for continuous variables. Bivariate between-group comparisons were performed using the chi-square test for categorical variables and the student’s t-test or Kruskal–Wallis test for continuous variables. The association between the PRS and blood magnesium levels in the second and third follow-ups was assessed using non-parametric Spearman correlation.

For both cross-sectional and prospective analyses, multivariable models were conducted using logistic regression for categorical variables and the results were expressed as Odds ratio (OR) and (95% confidence interval). For cross-sectional analyses, multivariable models were also conducted using analysis of variance for continuous variables and the results were expressed as adjusted average ± standard error. Multivariable models were adjusted for gender (man, woman), age (continuous), educational level (low, middle, high), smoking categories (never, former, current), born in Switzerland (yes, no), BMI categories (normal, overweight, obese), alcohol consumption (none, 1–13, 14–27, and 28 + units/week), AHEI (continuous), hypertension (yes, no), diabetes (yes, no), presence of sleep medication (yes, no), and other vitamin/mineral supplements (yes, no).

Two sensitivity analyses were conducted. The first one included only participants not on sleep medications. The second used inverse probability weighting (IPW) as indicated previously [33]. Briefly, logistic regression was used to estimate the likelihood of being included for each participant, and the inverse of predicted probability was then used for the multivariable analyses. In the multivariable analysis, correction for multiple testing was performed for each follow-up / method using the Benjamini–Hochberg procedure. Statistical significance was considered for a two-sided test with p < 0.05.

## Results

### Sample characteristics

During the first follow-up (2009–2012), 5,064 participants were eligible, of which 1,777 were excluded. In the second follow-up (2014–2017), 1,916 participants were included, while 2,965 were excluded. Finally, in the third follow-up (2018–2021), 1,561 participants were included and 2,190 were excluded. The reasons for exclusion are summarized in Supplementary Fig. 1. Supplementary Table 4 lists the characteristics of both included and excluded participants. Excluded participants were more likely to be women, older, had lower educational levels, and were less often born in Switzerland than included participants. Additionally, excluded participants had lower alcohol consumption, higher BMI, higher rates of diabetes, hypertension, and generally had a greater need for lipid-lowering treatments.

Table 1 summarizes the characteristics of magnesium supplement users in the first, second, and third follow-ups. In all surveys, supplement users were more likely to be female, older, and took other vitamin/mineral supplements and medications more frequently. Inconsistent differences (i.e.,significant in the first follow-up but not in the others) regarding being born in Switzerland, BMI levels and hypolipidemic treatment were found, while no differences were found for the other characteristics, including blood magnesium levels and PRS for magnesium levels.

**Table 1:** characteristics of magnesium supplement users, by follow-up period, CoLaus|PsyCoLaus study, Lausanne, Switzerland

	First (2009–2012)			Second (2014–2017)			Third (2018–2021)		
	Non-users	Users	*P*-value	Non-users	Users	*P*-value	Non-users	Users	*P*-value
Sample size	3712	175		1765	151		1444	117	
Female (%)	1928 (51.9)	117 (66.9)	< 0.001	859 (48.7)	94 (62.3)	0.001	721 (49.9)	78 (66.7)	< 0.001
Age (years)	57.0 ± 10.3	61.5 ± 10.4	< 0.001	60.6 ± 9.3	62.6 ± 9.5	0.011	63.0 ± 8.8	65.2 ± 9.9	0.010
*Educational level (%)*			0.212			0.122			0.809
High	846 (22.8)	35 (20.0)		462 (26.2)	36 (23.8)		371 (25.7)	32 (27.4)	
Middle	1008 (27.2)	58 (33.1)		493 (27.9)	54 (35.8)		436 (30.2)	37 (31.6)	
Low	1858 (50.1)	82 (46.9)		810 (45.9)	61 (40.4)		637 (44.1)	48 (41.0)	
Born in Switzerland (%)	2425 (65.3)	127 (72.6)	0.049	1198 (67.9)	104 (68.9)	0.801	976 (67.6)	87 (74.4)	0.131
*Smoking status (%)*			0.279			0.125			0.148
Never	1541 (41.5)	66 (37.7)		747 (42.3)	56 (37.1)		614 (42.5)	51 (43.6)	
Former	1390 (37.5)	76 (43.4)		693 (39.3)	72 (47.7)		571 (39.5)	53 (45.3)	
Current	781 (21.0)	33 (18.9)		325 (18.4)	23 (15.2)		259 (17.9)	13 (11.1)	
*Alcohol consumer (%)*			0.302			0.922			0.185
Never	814 (21.9)	49 (28)		342 (20.1)	31 (21.2)		354 (24.7)	33 (28.5)	
1–13 units/week	2314 (62.3)	102 (58.3)		1121 (65.7)	92 (63)		847 (59.2)	71 (61.2)	
14–27 units/week	464 (12.5)	19 (10.9)		204 (12)	19 (13)		186 (13)	12 (10.3)	
28 + units/week	120 (3.2)	5 (2.9)		39 (2.3)	4 (2.7)		44 (3.1)	0 (0)	
AHEI	31.8 ± 10.0	33.7 ± 10.8	0.018	32.1 ± 10	33.3 ± 9.2	0.187	31.6 ± 9.9	33.7 ± 9.8	0.032
BMI (kg/m^2^)	26.0 ± 4.4	25.4 ± 3.8	0.048	26.1 ± 4.3	25.7 ± 4.4	0.326	26.2 ± 4.4	25.4 ± 4.3	0.060
*BMI categories (%)*			0.363			0.335			0.503
Normal	1664 (44.8)	83 (47.4)		766 (43.4)	74 (49.0)		635 (44)	58 (49.6)	
Overweight	1454 (39.2)	71 (40.6)		723 (41.0)	53 (35.1)		548 (38)	40 (34.2)	
Obese	594 (16.0)	21 (12.0)		276 (15.6)	24 (15.9)		261 (18.1)	19 (16.2)	
Hypertension (%)	1498 (40.4)	80 (45.7)	0.152	706 (40.0)	67 (44.4)	0.293	687 (47.6)	50 (42.7)	0.313
Diabetes (%)	354 (9.5)	18 (10.3)	0.742	127 (7.2)	16 (10.6)	0.127	108 (7.5)	8 (6.8)	0.799
Hypolipidemic treatment (%)	720 (19.4)	52 (29.7)	0.001	353 (20)	34 (22.5)	0.460	288 (19.9)	25 (21.4)	0.712
Sleep medications (%)	268 (7.2)	23 (13.1)	0.004	122 (6.9)	18 (11.9)	0.023	88 (6.1)	15 (12.8)	0.005
Blood magnesium (mmol/L)	NA	NA		0.85 ± 0.06	0.85 ± 0.08	0.521	0.85 ± 0.06	0.86 ± 0.06	0.673
Magnesium PRS §	4.0 ± 1.7	4.1 ± 1.6	0.659	3.9 ± 1.7	3.8 ± 1.6	0.342	4.0 ± 1.7	4.0 ± 1.6	0.977
Other VMS (%)	567 (15.3)	74 (42.3)	< 0.001	393 (22.3)	70 (46.4)	< 0.001	328 (22.7)	58 (49.6)	< 0.001

AHEI, alternative healthy eating index; BMI, body mass index; NA, not available; PRS; polygenic risk score; VMS, vitamin/mineral supplements. Results are expressed as number of participants (column percentage) for categorical variables and as average ± standard deviation for continuous variables. Between-group comparisons performed using chi-square for categorical variables and student’s t-test for continuous variables. §: total sample size: 3178, 1562, and 1233 for the first, second and third follow-ups, respectively

### Cross-sectional associations between magnesium supplementation and self-reported sleep quality

The associations between magnesium supplement use and self-reported sleep parameters are summarized in Tables 2 and 3. In the bivariate analyses, magnesium supplement users reported a lower sleep quality and a higher frequency of night cramps in all surveys, and a higher prevalence of restless leg syndrome in the first and third follow-ups (Table 2). The multivariable-adjusted analyses showed that magnesium users tended to present a higher likelihood of night cramps and restless leg syndrome (Table 3). Analyses excluding participants taking sleep medicines or using IPW analysis confirmed the association between magnesium use and night cramps, but a lesser trend towards a higher likelihood or restless leg syndrome (Supplementary Table 5). No association was found between magnesium use and Epworth, sleep quality, risk of sleep apnoea or sleep disordered breathing (Table 3 and Supplementary Table 5).

**Table 2:** bivariate associations between magnesium supplement use and self-reported sleep quality, by follow-up period, CoLaus|PsyCoLaus study, Lausanne, Switzerland

	First (2009–2012)			Second (2014–2017)			Third (2018–2021)		
	Non-users	Users	*P*-value	Non-users	Users	*P*-value	Non-users	Users	*P*-value
Sample size	3712	175		1765	151		1444	117	
Pittsburgh global score	4 [3–7]	5 [3–8]	0.019	4 [3–6]	5 [3–7]	0.011	4 [2–6]	5 [3–8]	0.004
PSQI > 5 (%)	1272 (34.3)	76 (43.4)	0.013	566 (32.1)	60 (39.7)	0.054	432 (29.9)	49 (41.9)	0.007
Epworth score	5 [3–8]	5 [3–7]	0.078	5 [3–8]	5 [3–8]	0.635	5 [3–8]	4 [3–7]	0.329
Positive Epworth (%)	407 (11.0)	16 (9.1)	0.450	179 (10.1)	18 (11.9)	0.490	137 (9.5)	9 (7.7)	0.521
High risk of sleep apnoea (%)	833 (23.7)	40 (24.5)	0.814	342 (21.1)	38 (28.8)	0.040	258 (20.4)	21 (21.9)	0.723
NoSAS score *	7 [4–11]	7 [4–9]	0.520	–	–	–	7 [5–11]	7 [4–9]	0.125
Sleep-disordered breathing (%)*	679 (41.8)	34 (37.0)	0.363	–	–	–	621 (49.2)	42 (44.7)	0.397
STOP score	2 [2, 3]	2 [1–3]	0.848	2 [1–3]	2 [1–3]	0.917	2 [1–3]	2 [1–3]	0.807
Positive STOP category (%)	2414 (77.0)	107 (74.8)	0.551	1076 (74.7)	84 (73.7)	0.806	836 (72.7)	64 (70.3)	0.626
Restless leg syndrome (%)	471 (12.7)	43 (24.6)	< 0.001	228 (57.0)	28 (59.6)	0.736	174 (12.1)	25 (21.4)	0.004
Night cramps 1 + /week (%)	161 (4.4)	32 (18.6)	< 0.001	83 (5.0)	17 (11.9)	0.001	-	–	–

PSQI, Pittsburgh Sleep Quality Index. *, n = 1626 non-users and 92 users for the first follow-up. Results are expressed as number of participants (column percentage) for categorical variables and as median [interquartile range] for continuous variables. Between-group comparisons performed using chi-square for categorical variables and Kruskal–Wallis test for continuous variables

**Table 3:** multivariable-adjusted associations between magnesium supplement use and self-reported sleep quality, by follow-up period, CoLaus|PsyCoLaus study, Lausanne, Switzerland

	First (2009–2012)			Second (2014–2017)			Third (2018–2021)		
	No	Yes	*P*-value	No	Yes	*P*-value	No	Yes	*P*-value
Pittsburgh global score	4.9 ± 0.1	5.4 ± 0.2	0.405	4.8 ± 0.1	5.0 ± 0.2	0.898	4.6 ± 0.1	5.1 ± 0.3	0.432
PSQI > 5	1 (ref)	1.23 (0.88–1.73)	0.994	1 (ref)	1.10 (0.75–1.60)	0.898	1 (ref)	1.37 (0.89–2.10)	0.851
Epworth score	5.9 ± 0.1	5.8 ± 0.3	0.994	5.7 ± 0.1	5.6 ± 0.3	0.898	5.6 ± 0.1	5.4 ± 0.3	0.851
Positive Epworth	1 (ref)	1.06 (0.62–1.83)	0.994	1 (ref)	1.37 (0.80–2.35)	0.898	1 (ref)	0.93 (0.45–1.94)	0.851
High risk of sleep apnoea	1 (ref)	1.10 (0.72–1.69)	0.994	1 (ref)	1.57 (0.97–2.54)	0.520	1 (ref)	1.30 (0.71–2.38)	0.851
NoSAS score *	7.1 ± 0.1	7.0 ± 0.2	0.994	–	–		8.1 ± 0.1	7.9 ± 0.2	0.851
Sleep-disord. breathing *	1 (ref)	0.76 (0.36–1.60)	0.994	–	–		1 (ref)	1.76 (0.87–3.55)	0.851
STOP score	2.1 ± 0.1	2.1 ± 0.1	0.994	2.1 ± 0.1	2.0 ± 0.1	0.898	2.0 ± 0.1	2.1 ± 0.1	0.851
Positive STOP category	1 (ref)	1.00 (0.66–1.51)	0.994	1 (ref)	1.06 (0.66–1.69)	0.898	1 (ref)	1.15 (0.68–1.92)	0.851
Restless leg syndrome	1 (ref)	1.99 (1.37–2.90)	0.003	1 (ref)	1.13 (0.58–2.18)	0.898	1 (ref)	1.85 (1.13–3.03)	0.140
Night cramps	1 (ref)	4.24 (2.70–6.64)	3.4E-9	1 (ref)	2.29 (1.27–4.14)	0.054	–	–	–

PSQI, Pittsburgh Sleep Quality Index. *, n = 1626 non-users and 92 users for the first follow-up. Results are expressed as odds ratio and (95% confidence interval) for categorical variables and as multivariable-adjusted mean ± standard error. Statistical analysis using logistic regression for categorical variables and analysis of variance for continuous variables. Multivariable models adjusted for gender (man, woman), age (continuous), educational level (low, middle, high), smoking categories (never, former, current), born in Switzerland (yes, no), BMI categories (normal, overweight, obese), alcohol consumption (none, 1–13, 14–27, and 28 + units/week), AHEI (continuous), hypertension (yes, no), diabetes (yes, no), other vitamin/mineral supplements (yes, no) and presence of sleep medication (yes, no). In the multivariable analysis, correction for multiple testing was performed for each follow-up using the Benjamini–Hochberg procedure

### Cross-sectional associations between magnesium supplementation and objective sleep parameters

The associations between magnesium supplement use and objectively reported sleep parameters are summarized in Table 4. The bivariate and multivariable-adjusted analyses showed no significant difference in objectively assessed sleep parameters between magnesium supplement users and non-users, including PLMS and PLMSI.

**Table 4:** Bivariate and multivariable analysis of the association between magnesium supplement use and objectively assessed sleep, HypnoLaus study, Lausanne, Switzerland

	Bivariate		*P*-value	Multivariable		*P*-value
	Non-users	Users		Non-users	Users	
Sleep onset (minutes)	11 [5–21]	10 [4–22]	§ 0.667	17.7 ± 0.5	15 ± 2.2	0.966
Total sleep time (minutes)	401 ± 72	406 ± 71	0.482	401 ± 2	407 ± 7	0.966
Sleep efficiency (%)	84.7 ± 10.8	83.4 ± 11.5	0.214	84.6 ± 0.2	84.7 ± 0.9	0.966
Number of awakenings	22 [16–31]	23 [17–34]	§ 0.162	25 ± 0.3	26 ± 1.2	0.966
*% of sleep time spent in*						
Stage 1 sleep	10.2 [7.2–14.7]	10.1 [7.4–16.0]	§ 0.519	11.9 ± 0.2	12.6 ± 0.6	0.966
Stage 2 sleep	45.6 [39.7–52.3]	46.2 [39.7–55.5]	§ 0.363	46.4 ± 0.2	46.3 ± 0.9	0.966
Slow wave sleep	19.3 [14.1–25.0]	19.2 [12.9–24.5]	§ 0.244	19.8 ± 0.2	19.2 ± 0.8	0.966
REM sleep	22.4 [18.2–26.0]	20.9 [16.2–25.7]	§ 0.096	21.9 ± 0.1	21.8 ± 0.6	0.966
*Periodic limb movements*						
Number	0 [0–19]	2 [0–20]	§ 0.743	17.9 ± 0.8	18.1 ± 3.4	0.966
Index	0 [0–2.9]	0.4 [0–2.9]	§ 0.819	2.7 ± 0.1	2.5 ± 0.5	0.966

For bivariate analysis, results are expressed as average ± standard deviation or as median [interquartile range] and between-group comparisons performed using student’s t-test or Kruskal–Wallis test (§). For multivariable analysis, results are expressed as adjusted mean ± standard error, and comparisons were performed using analysis of variance adjusting for gender (man, woman), age (continuous), educational level (low, middle, high), smoking categories (never, former, current), born in Switzerland (yes, no), BMI categories (normal, overweight, obese), alcohol consumption (none, 1–13, 14–27, and 28 + units/week), hypertension (yes, no), diabetes (yes, no), other vitamin/mineral supplements (yes, no) and presence of sleep medication (yes, no). In the multivariable analysis, correction for multiple testing was performed using the Benjamini–Hochberg procedure

### Cross-sectional associations between the polygenic risk score and nocturnal cramps/ restless leg syndrome

The distribution of the magnesium PRS for the first, second and third follow-ups is depicted in Supplementary Fig. 2. A symmetrical, gaussian-like distribution was observed in all cases. The PRS was positively associated with magnesium levels, Spearman correlation coefficients of 0.169 and 0.129 for the second and third follow-ups, both p < 0.001. The bivariate and multivariate analyses of the magnesium PRS between participants with and without cramps showed no significant differences in either the first or second follow-ups (Table 5).

**Table 5:** bivariate and multivariable analysis of the magnesium genetic score between participants with and without cramps, by follow-up period, CoLaus|PsyCoLaus study, Lausanne, Switzerland

	Without cramps	With cramps	*P*-value
*First (2009–2012)*			
Bivariate	4.0 ± 1.7	3.9 ± 1.6	0.610
Multivariable	4.00 ± 0.03	3.96 ± 0.14	0.804
*Second (2014–2017)*			
Bivariate	3.9 ± 1.7	3.8 ± 1.7	0.627
Multivariable	3.93 ± 0.05	3.87 ± 0.2	0.768

For bivariate analysis, results are expressed as average ± standard deviation and between-group comparisons performed using student’s t-test. For multivariable analysis, results are expressed as adjusted mean ± standard error, and comparisons were performed using analysis of variance adjusting for gender (man, woman), age (continuous), educational level (low, middle, high), smoking categories (never, former, current), born in Switzerland (yes, no), BMI categories (normal, overweight, obese), alcohol consumption (none, 1–13, 14–27, and 28 + units/week), hypertension (yes, no), diabetes (yes, no), other vitamin/mineral supplements (yes, no) and presence of sleep medication (yes, no)

### Prospective associations between magnesium supplementation and incidence of nocturnal cramps/ restless leg syndrome

During the first follow-up (2009–2012), 5,064 participants were eligible, of which 2,298 were excluded for the analyses on night cramps. Of the 2,766 participants included, 117 (4.2%) took magnesium supplements at baseline. After a median follow-up time of 5.3 years (interquartile range; 5.1–5.4 years), 117 (4.2%) of the participants presented with incident night cramps. Participants taking magnesium supplements at baseline had a higher incidence of night cramps (10.3% vs. 4.0%, p = 0.001), and this difference persisted after multivariable adjustment: OR and 95% confidence interval: 2.02 (1.02–3.99). Similar findings were obtained after excluding participants taking sleep medications: OR 2.08 (1.01–4.26).

For incidence of RLS, 3,993 participants were excluded. Among the 1,071 participants included, 54 (5%) took magnesium supplements and 241 (22.5%) had incident RLS. No difference was found between participants taking magnesium supplements and participants not taking them (22.1% vs. 29.6%, p = 0.198) and similar results were obtained after multivariable adjustment: OR 1.42 (0.73–2.76) p = 0.298 and after excluding participants taking sleep medications: OR 0.95 (0.42–2.12) p = 0.900.

## Discussion

In the present observational study, participants taking magnesium supplements did not show significantly better subjective or objective sleep parameters. Indeed, the observed benefits from taking these kinds of supplements were broadly inconsistent across the three follow-ups and different outcomes.

### Cross-sectional associations between magnesium supplementation and subjective sleep parameters

Based on the analysis of self-reported sleep quality, participants taking magnesium supplements presented with a higher prevalence of RLS, and night cramps. This finding was unexpected and against our initial hypothesis. It also contradicted previous research, which generally concluded that while magnesium does not significantly improve sleep disorders, it also does not appear to worsen them [12, 13, 34]. The likely explanation for these inconsistencies in results across various studies is a reverse causation bias: individuals experiencing more frequent nocturnal cramps are more likely to take magnesium supplements, as it is largely recommended in the lay media despite absence of scientific evidence [35]. However, if magnesium supplementation was effective in alleviating cramps, one would expect these individuals to report the condition less frequently. Further, no differences in blood magnesium levels were found between supplement users and non-users, suggesting that magnesium levels are not associated with sleep disorders, RLS, or night cramps. A possible explanation is that additional magnesium intake is rapidly regulated by the intestine and the kidneys, to maintain blood magnesium levels within a specified range [36].

### Cross-sectional associations between magnesium supplementation and objective sleep parameters

The polysomnography results showed no significant differences in objectively measured sleep parameters or sleep disorders between individuals who used magnesium supplements and those who did not. This aligns with most previous studies, which reported similar findings [7, 9, 10, 12]. However, one study [37] reported conflicting results regarding magnesium's effects on sleep quality, while only a few others observed a measurable impact [5, 6]. Importantly, no association between PLMS and PLMSI and magnesium supplement use was found, a finding somewhat in contradiction with the subjective information on RLS or night cramps. Given that sleep is influenced by multiple factors, the lack of observed benefits from magnesium supplementation could be attributed to several reasons. Variations in study methodologies, participant characteristics, or dosage may explain the inconsistent findings. Additionally, not all forms of magnesium have the same bioavailability, meaning absorption rates can differ. Another key consideration is that if participants already have adequate magnesium levels, supplementation may not provide additional benefits for sleep. As objectively measured sleep parameters are more reliable than self-reported data and did not show any difference between users and no users, we conclude that magnesium supplementation does not appear to have a significant effect on sleep quality or sleep duration. However, further studies with more standardized methodologies could provide clearer insights.

Additionally, we sought to determine whether specific SNPs associated with lower magnesium levels were linked to a higher prevalence of nocturnal cramps and RLS. To our knowledge, no prior research has explored this connection between genetics, magnesium levels, and sleep parameters. Our findings indicated that the magnesium-related PRS did not differ significantly between individuals with and without cramps across study periods. These results suggest that genetically determined magnesium levels are not associated with the prevalence of cramps.

### Prospective associations between magnesium supplementation and incidence of nocturnal cramps/ restless leg syndrome

After five years of follow-up, participants taking magnesium supplements had a higher likelihood of incident night cramps, while no differences in incidence of RLS were found. The higher incidence of night cramps among participants taking magnesium supplements was unexpected. As we failed to find any prospective study assessing similar associations, our findings should be interpreted with caution. A possible explanation is that people prone to night cramps take magnesium supplements more frequently as a preventive measure. Indeed, participants taking magnesium supplements tended to report more frequently night cramps (supplementary Fig. 3). Hence, in this case, magnesium supplement consumption would act as a marker of predisposition to night cramps, rather than a preventive measure. Interestingly, among the 117 participants taking magnesium supplements at the first follow-up, 54 (46%) continued to do so at the second follow-up. As this group also presented with the highest incidence of night cramps (13%), our results suggest that long-term consumption of magnesium supplements is ineffective in preventing night cramps.

### Implications for clinical practice

Our results confirm the lack of association between magnesium supplements and both subjective and objective sleep parameters. As a result, and in agreement with other authors [7, 9, 10] we cannot recommend supplementation with magnesium for sleep disorders, RLS, or night cramps.

### Strengths and limitations

The biggest strength of this study is its big sample size relative to other studies focusing on magnesium supplements and sleep parameters. This study also used both subjectively and objectively assessed sleep parameters, and, to our knowledge, is the first one to explore the connection between genetics, magnesium levels, and sleep parameters. It is also the first one to prospectively assess the association between magnesium supplementation and incidence of RLS or night cramps.

The primary limitation of this study lies in its observational nature. Participants chose to take magnesium supplements at their own will, both in terms of usage and duration. Consequently, those who opted for such supplementation are likely individuals who are generally more health-conscious, with a healthier diet [38]. Still, no difference in blood magnesium levels was found between magnesium consumers and non-consumers was found, suggesting that neither the supplements nor the healthier diet influenced blood magnesium levels. Conducting a randomized, double-blind, placebo-controlled trial would be valuable to determine whether the findings align with our results and those of previous studies. Another major limitation of this study is that participants often bought their magnesium supplements at supermarkets, making it difficult to assess the exact dosage [39]. Hence, it was not possible to perform dose–response analyses or to account for potential differences in bioavailability [40], which may influence the effectiveness of magnesium supplements Only total blood magnesium levels were available, and it would have been more interesting to measure ionized or intracellular magnesium; still, previous studies have shown that total and ionized magnesium are strongly correlated (r > 0.8) [41]. Finally, a sizable fraction of the initial sample had to be excluded due to missing data or non-participation in the follow-up. Although we tried to correct for this selection bias using inverse probability weighting, it is likely that residual confounding remains.

## Conclusion

In this observational study, we found no consistent association between magnesium supplementation and subjective/or objective sleep parameters or RLS. Magnesium supplementation did not prevent night cramps. The higher incidence of night cramps among magnesium supplement users merits further investigation.

## Supplementary Information

Below is the link to the electronic supplementary material.

Supplementary Material 1

Supplementary Material 2

Supplementary Material 3

Supplementary Material 5

## Data Availability

The data of CoLaus|PsyCoLaus study used in this article cannot be fully shared as they contain potentially sensitive personal information on participants. According to the Ethics Committee for Research of the Canton of Vaud, sharing these data would be a violation of the Swiss legislation with respect to privacy protection. However, coded individual-level data that do not allow researchers to identify participants are available upon request to researchers who meet the criteria for data sharing of the CoLaus|PsyCoLaus Datacenter (CHUV, Lausanne, Switzerland). Any researcher affiliated to a public or private research institution who complies with the CoLaus|PsyCoLaus standards can submit a research application to research.colaus@chuv.ch or research.psycolaus@chuv.ch. Proposals will be evaluated by the Scientific Committee (SC) of the CoLaus|PsyCoLaus study. Detailed instructions for gaining access to the CoLaus|PsyCoLaus data used in this study are available at https://www.colaus-psycolaus.ch/professionals/how-to-collaborate/.
