# Supplementary material for: Magnesium supplements, sleep quality, and nocturnal leg cramps: a combination of cross-sectional and prospective studies
Source: Eur J Nutr. 2026 Feb 16;65(2):60. doi: 10.1007/s00394-026-03910-2 (PMC13661682; doi:10.1007/s00394-026-03910-2)
Supplement: Supplementary file 1 [file 394_2026_3910_MOESM4_ESM.docx]

**Supplementary information**

**Supplementary table 1:** Categorization of the NoSAS score

|  | **Item** | **Scoring** |
| --- | --- | --- |
| **N** | **N**eck circumference >40 cm | 4 |
| **O** | **O**verweight: BMI ≥ 25 and <30 kg/m^2^ | 3 |
| **O** | **O**besity: BMI ≥ 30 kg/m^2^ | 5 |
| **S** | Male **s**ex | 2 |
| **A** | **A**ge >55 years | 4 |
| **S** | **S**noring | 2 |
|  | **Range** | **0-17** |

Note: chose either overweight or obesity.

**Supplementary table 2.** The four restless legs syndrome (RLS) questions defined by the 1995 International RLS study group

| **#** | **Item** |
| --- | --- |
| **1** | An urge to move the legs, usually accompanied or caused by uncomfortable and unpleasant sensations in the legs (Sometimes the urge to move is present without the uncomfortable sensations and sometimes the arms or other body parts are involved in addition to the legs) |
| **2** | The urge to move or unpleasant sensations begin or worsen during periods of rest or inactivity such as lying or sitting |
| **3** | The urge to move or unpleasant sensations are partially or totally relieved by movement, such as walking or stretching, at least as long as the activity continues |
| **4** | The urge to move or unpleasant sensations are worse in the evening or night than during the day or only occur in the evening or night (When symptoms are very severe, the worsening at night may not be noticeable but must have been previously present) |

**Supplementary table 3**: single nucleotide polymorphisms used to construct the magnesium genetic score.

| **SNP** | **Chr.** | **Position** | **Risk allele** | **Mapped genes** |
| --- | --- | --- | --- | --- |
| rs4072037 | 1 | 155,162,067 | C | *MUC1*: mucin 1, cell surface associated |
| rs4460629 | 1 | 155,135,335 | T | *HMGN2P18*: high mobility group nucleosomal binding domain 2 pseudogene 18;  *KRTCAP2*: keratinocyte associated protein 2 |
| rs13146355 | 4 | 77,412,140 | G | *SHROOM3*: shroom family member 3 |
| rs11144134 | 9 | 77,499,796 | T | *TRPM6:* transient receptor potential cation channel subfamily M member 6 |
| rs3925584 | 11 | 30,760,335 | C | *DCDC1*: doublecortin domain containing 1;  *MPPED2-AS1*: MPPED2 antisense RNA 1 |

Chr, chromosome; SNP, single nucleotide polymorphism.

**Supplementary table 4**: characteristics of included and excluded participants, by study period, CoLaus|PsyCoLaus study, Lausanne, Switzerland.

|  | **First (2009-2012)** | | | **Second (2014-2017)** | | | **Third (2018-2021)** | | |
| --- | --- | --- | --- | --- | --- | --- | --- | --- | --- |
|  | **Included** | **Excluded** | **P-value** | **Included** | **Excluded** | **P-value** | **Included** | **Excluded** | **P-value** |
| Sample size | 3887 | 1777 |  | 1916 | 2965 |  | 1561 | 2190 |  |
| Female (%) | 2045 (52.6) | 662 (56.2) | 0.029 | 953 (49.7) | 1736 (58.6) | <0.001 | 799 (51.2) | 1284 (58.6) | <0.001 |
| Age (years) | 57.2 ± 10.4 | 59.6 ± 10.8 | <0.001 | 60.8 ± 9.4 | 64.3 ± 10.9 | <0.001 | 63.1 ± 8.9 | 66.8 ± 10.3 | <0.001 |
| Educational level (%) |  |  | <0.001 |  |  | <0.001 |  |  | <0.001 |
| High | 881 (22.7) | 198 (16.9) |  | 498 (26.0) | 533 (18.0) |  | 403 (25.8) | 443 (20.3) |  |
| Middle | 1066 (27.4) | 240 (20.5) |  | 547 (28.6) | 711 (24.0) |  | 473 (30.3) | 552 (25.2) |  |
| Low | 1940 (49.9) | 734 (62.6) |  | 871 (45.5) | 1717 (58.0) |  | 685 (43.9) | 1193 (54.5) |  |
| Born in Switzerland (%) | 2552 (65.7) | 632 (53.7) | <0.001 | 1302 (68) | 1760 (59.4) | <0.001 | 1063 (68.1) | 1313 (60.0) |  |
| Smoking status (%) |  |  | 0.025 |  |  | 0.368 |  |  | 0.087 |
| Never | 1607 (41.3) | 428 (38.2) |  | 803 (41.9) | 1083 (42.0) |  | 665 (42.6) | 830 (45.3) |  |
| Former | 1466 (37.7) | 417 (37.2) |  | 765 (39.9) | 989 (38.3) |  | 624 (40.0) | 732 (39.9) |  |
| Current | 814 (20.9) | 275 (24.6) |  | 348 (18.2) | 507 (19.7) |  | 272 (17.4) | 272 (14.8) |  |
| Alcohol consumer (%) |  |  | <0.001 |  |  | <0.001 |  |  | <0.001 |
| Never | 863 (22.2) | 418 (35.5) |  | 373 (20.1) | 802 (33.6) |  | 387 (25) | 403 (32.3) |  |
| 1-13 units/week | 2416 (62.2) | 595 (50.6) |  | 1213 (65.5) | 1275 (53.4) |  | 918 (59.3) | 697 (55.8) |  |
| 14-27 units/week | 483 (12.4) | 133 (11.3) |  | 223 (12) | 250 (10.5) |  | 198 (12.8) | 121 (9.7) |  |
| 28+ units/week | 125 (3.2) | 31 (2.6) |  | 43 (2.3) | 61 (2.6) |  | 44 (2.8) | 28 (2.2) |  |
| BMI (kg/m^2^) | 26.0 ± 4.4 | 26.9 ± 5.3 | <0.001 | 26.0 ± 4.3 | 26.7 ± 5.0 | <0.001 | 26.1 ± 4.4 | 26.5 ± 4.9 | 0.004 |
| BMI categories (%) |  |  | <0.001 |  |  | <0.001 |  |  | 0.037 |
| Normal | 1747 (44.9) | 433 (38.9) |  | 840 (43.8) | 1017 (39.7) |  | 693 (44.4) | 808 (40.6) |  |
| Overweight | 1525 (39.2) | 435 (39.1) |  | 776 (40.5) | 999 (39.0) |  | 588 (37.7) | 768 (38.6) |  |
| Obese | 615 (15.8) | 244 (21.9) |  | 300 (15.7) | 549 (21.4) |  | 280 (17.9) | 412 (20.7) |  |
| Hypertension (%) | 1578 (40.6) | 518 (44.4) | 0.021 | 773 (40.3) | 1490 (53.8) | <0.001 | 737 (47.2) | 1124 (54.2) | <0.001 |
| Diabetes (%) | 372 (9.6) | 167 (14.4) | <0.001 | 143 (7.5) | 355 (13.5) | <0.001 | 116 (7.4) | 267 (13.3) | <0.001 |
| Hypolipidemic treatment (%) | 772 (19.9) | 284 (24.1) | 0.002 | 387 (20.2) | 764 (25.8) | <0.001 | 313 (20.1) | 489 (22.3) | 0.094 |
| Sleep medications (%) | 291 (7.5) | 142 (12.1) | <0.001 | 140 (7.3) | 356 (12.0) | <0.001 | 103 (6.6) | 196 (9.0) | 0.009 |
| Other VMS (%) | 641 (16.5) | 178 (15.1) | 0.264 | 463 (24.2) | 823 (27.8) | 0.005 | 386 (24.7) | 559 (25.5) | 0.579 |

BMI, body mass index; VMS; vitamin/mineral supplements. Results are expressed as number of participants (column percentage) for categorical variables and as average ± standard deviation for continuous variables. Between-group comparisons performed using chi-square for categorical variables and student’s t-test for continuous variables.

**Supplementary table 5:** multivariable-adjusted associations between magnesium supplement use and self-reported sleep quality, by study period, CoLaus|PsyCoLaus study, Lausanne, Switzerland. Analyses excluding sleep medication or using inverse probability weighting.

|  | **First (2009-2012)** | | | **Second (2014-2017)** | | | **Third (2018-2021)** | | |
| --- | --- | --- | --- | --- | --- | --- | --- | --- | --- |
|  | **No** | **Yes** | **P-value** | **No** | **Yes** | **P-value** | **No** | **Yes** | **P-value** |
| **Excluding sleep medication** |  |  |  |  |  |  |  |  |  |
| Pittsburgh global score | 4.6 ± 0.1 | 4.9 ± 0.2 | 0.906 | 4.5 ± 0.1 | 4.6 ± 0.2 | 0.997 | 4.3 ± 0.1 | 4.9 ± 0.3 | 0.470 |
| PSQI >5 | 1 (ref) | 1.12 (0.79 - 1.60) | 0.906 | 1 (ref) | 1.05 (0.70 - 1.56) | 0.997 | 1 (ref) | 1.40 (0.89 - 2.18) | 0.918 |
| Epworth score | 5.9 ± 0.1 | 5.8 ± 0.3 | 0.906 | 5.7 ± 0.1 | 5.7 ± 0.3 | 0.997 | 5.6 ± 0.1 | 5.5 ± 0.4 | 0.918 |
| Positive Epworth | 1 (ref) | 1.06 (0.60 - 1.9) | 0.906 | 1 (ref) | 1.40 (0.79 - 2.48) | 0.997 | 1 (ref) | 0.96 (0.44 - 2.09) | 0.918 |
| Hight risk of sleep apnoea | 1 (ref) | 0.97 (0.6 - 1.56) | 0.906 | 1 (ref) | 1.86 (1.12 - 3.09) | 0.136 | 1 (ref) | 1.13 (0.58 - 2.21) | 0.918 |
| NoSAS score * | 7.1 ± 0.1 | 7.0 ± 0.2 | 0.906 | - | - |  | 8.1 ± 0.1 | 7.9 ± 0.2 | 0.918 |
| Sleep-disord. breathing * | 1 (ref) | 0.70 (0.31 - 1.56) | 0.906 | - | - |  | 1 (ref) | 1.84 (0.88 - 3.86) | 0.848 |
| STOP score | 2.1 ± 0.1 | 2.1 ± 0.1 | 0.906 | 2.0 ± 0.1 | 2.1 ± 0.1 | 0.997 | 2.0 ± 0.1 | 2.1 ± 0.1 | 0.918 |
| Positive STOP category | 1 (ref) | 0.95 (0.62 - 1.45) | 0.906 | 1 (ref) | 1.29 (0.77 - 2.17) | 0.997 | 1 (ref) | 1.18 (0.69 - 2.03) | 0.918 |
| Restless leg syndrome | 1 (ref) | 1.88 (1.24 - 2.85) | 0.030 | 1 (ref) | 1.07 (0.54 - 2.15) | 0.997 | 1 (ref) | 1.66 (0.97 - 2.84) | 0.594 |
| Night cramps | 1 (ref) | 4.22 (2.57 - 6.92) | 1.3E-7 | 1 (ref) | 2.75 (1.47 - 5.13) | 0.009 | - | - | - |
| **Using IPW** |  |  |  |  |  |  |  |  |  |
| PSQI >5 | 1 (ref) | 1.27 (0.92 - 1.76) | 0.755 | 1 (ref) | 1.06 (0.72 - 1.57) | 0.846 | 1 (ref) | 1.56 (1.01 - 2.44) | 0.240 |
| Positive Epworth | 1 (ref) | 1.03 (0.60 - 1.78) | 0.923 | 1 (ref) | 1.45 (0.80 - 2.62) | 0.846 | 1 (ref) | 1.04 (0.46 - 2.32) | 0.932 |
| High risk of sleep apnoea | 1 (ref) | 1.08 (0.71 - 1.64) | 0.923 | 1 (ref) | 1.44 (0.86 - 2.39) | 0.815 | 1 (ref) | 1.42 (0.77 - 2.64) | 0.786 |
| Sleep-disord. breathing * | 1 (ref) | 0.75 (0.32 - 1.77) | 0.923 | - | - |  | 1 (ref) | 1.93 (0.95 - 3.96) | 0.284 |
| Positive STOP category | 1 (ref) | 0.98 (0.65 - 1.49) | 0.923 | 1 (ref) | 1.05 (0.64 - 1.72) | 0.846 | 1 (ref) | 1.19 (0.69 - 2.05) | 0.932 |
| Restless leg syndrome | 1 (ref) | 1.99 (1.35 - 2.92) | 0.003 | 1 (ref) | 1.23 (0.62 - 2.43) | 0.846 | 1 (ref) | 1.93 (1.17 - 3.17) | 0.060 |
| Night cramps | 1 (ref) | 4.43 (2.86 - 6.87) | 2.0E-10 | 1 (ref) | 2.06 (1.05 - 4.03) | 0.210 | - | - | - |

PSQI, Pittsburgh Sleep Quality Index. IPW, inverse probability weighting. *, n=1626 non-users and 92 users for the first follow-up. Results are expressed as odds ratio and (95% confidence interval) for categorical variables and as multivariable-adjusted mean ±standard error. Statistical analysis using logistic regression for categorical variables and analysis of variance for continuous variables. Multivariable models adjusted for gender (man, woman), age (continuous), educational level (low, middle, high), smoking categories (never, former, current), born in Switzerland (yes, no), BMI categories (normal, overweight, obese), alcohol consumption (none, 1-13, 14-27, and 28+ units/week), hypertension (yes, no), and diabetes (yes, no) and other vitamin/mineral supplements (yes, no). For analyses using inverse probability weighting, a further adjustment on presence of sleep medication (yes, no) was conducted. Correction for multiple testing was performed for each follow-up / method using the Benjamini-Hochberg procedure.
